# The 3 I’s of immunity and aging: immunosenescence, inflammaging, and immune resilience

**DOI:** 10.3389/fragi.2024.1490302

**Published:** 2024-10-16

**Authors:** Marianna V. Wrona, Rituparna Ghosh, Kaitlyn Coll, Connor Chun, Matthew J. Yousefzadeh

**Affiliations:** ^1^ Columbia University in the City of New York, New York, NY, United States; ^2^ Columbia Center for Human Longevity, Columbia University Medical Center, New York, NY, United States; ^3^ Columbia Center for Translational Immunology, Columbia University Medical Center, New York, NY, United States; ^4^ Department of Medicine, Columbia University Medical Center, New York, NY, United States; ^5^ Florida International University, Miami, FL, United States; ^6^ Bronx High School of Science, New York, NY, United States

**Keywords:** immunosenescence, inflammaging, cellular senescence, immune resilience, inflammation

## Abstract

As we age, our immune system’s ability to effectively respond to pathogens declines, a phenomenon known as immunosenescence. This age-related deterioration affects both innate and adaptive immunity, compromising immune function and leading to chronic inflammation that accelerates aging. Immunosenescence is characterized by alterations in immune cell populations and impaired functionality, resulting in increased susceptibility to infections, diminished vaccine efficacy, and higher prevalence of age-related diseases. Chronic low-grade inflammation further exacerbates these issues, contributing to a decline in overall health and resilience. This review delves into the characteristics of immunosenescence and examines the various intrinsic and extrinsic factors contributing to immune aging and how the hallmarks of aging and cell fates can play a crucial role in this process. Additionally, it discusses the impact of sex, age, social determinants, and gut microbiota health on immune aging, illustrating the complex interplay of these factors in altering immune function. Furthermore, the concept of immune resilience is explored, focusing on the metrics for assessing immune health and identifying strategies to enhance immune function. These strategies include lifestyle interventions such as diet, regular physical activity, stress management, and the use of gerotherapeutics and other approaches. Understanding and mitigating the effects of immunosenescence are crucial for developing interventions that support robust immune responses in aged individuals.

## Introduction

The immune system plays a crucial role in protecting our bodies from harmful pathogens. It is divided into two segments: innate immunity and adaptive immunity. The innate immune system acts as an immediate but non-specific first responder to defend against pathogens, composed of phagocytic and natural killer cells. Besides innate immune cells, another important component of the innate system includes physical barriers like skin and mucous membranes. Meanwhile, adaptive immunity is more specialized and requires time to mount a high-affinity and specific response, relying on anticipatory receptors that recognize pathogen-specific antigens. The adaptive immune response is centered around B and T lymphocytes, which are produced in the bone marrow and thymus, respectively (Farber, 2020; Lam et al., 2024). With age, the ability of our immune system to mount productive and timely responses to pathogens diminishes. This decline is known as immunosenescence and affects both innate and adaptive immunity (Liu et al., 2023). Another consequence of immunosenescence is the presence of chronic inflammation that develops over time (Pawelec et al., 2020).

This review highlights the various aspects and characteristics of immunosenescence, focusing on key immune cell types and the factors contributing to immune aging. It also examines Immune resilience by discussing the metrics used to assess immune health and function, along with interventions that can enhance immune resilience. This summary aims to provide a deeper understanding of the mechanisms behind immune aging and the strategies that can bolster immune function, ultimately promoting better health and longevity.

### Inflammaging and cellular senescence: sources of inflammation

Chronic stress and accumulated damage, whether occurring naturally or from acute and chronic infections, can lead to persistent inflammation, a precursor to altered cellular states known as the hallmarks of aging (Borgoni et al., 2021; Lopez-Otin et al., 2023). These hallmarks are known to accumulate with both chronological age (solely due to the passage of time) and biological age. While chronological age is exact, biological age is much more variable and can be impacted by factors like genetics, epigenetics, environment, and disease (Baker and Sprott, 1988). Thus, there is great interest in measuring biological age as a readout of both health but also for efficacy of interventions. Analysis of biological age can provide insights into the stochastic nature of aging and begin to understand how two organisms of the same species may be chronologically identical but have disparate phenotypes related to aging. Biological age endpoints typically involve measuring changes related to these pillars of aging as a surrogate endpoint for biological aging (Moqri et al., 2023; Liu et al., 2009).

**FIGURE 1:**
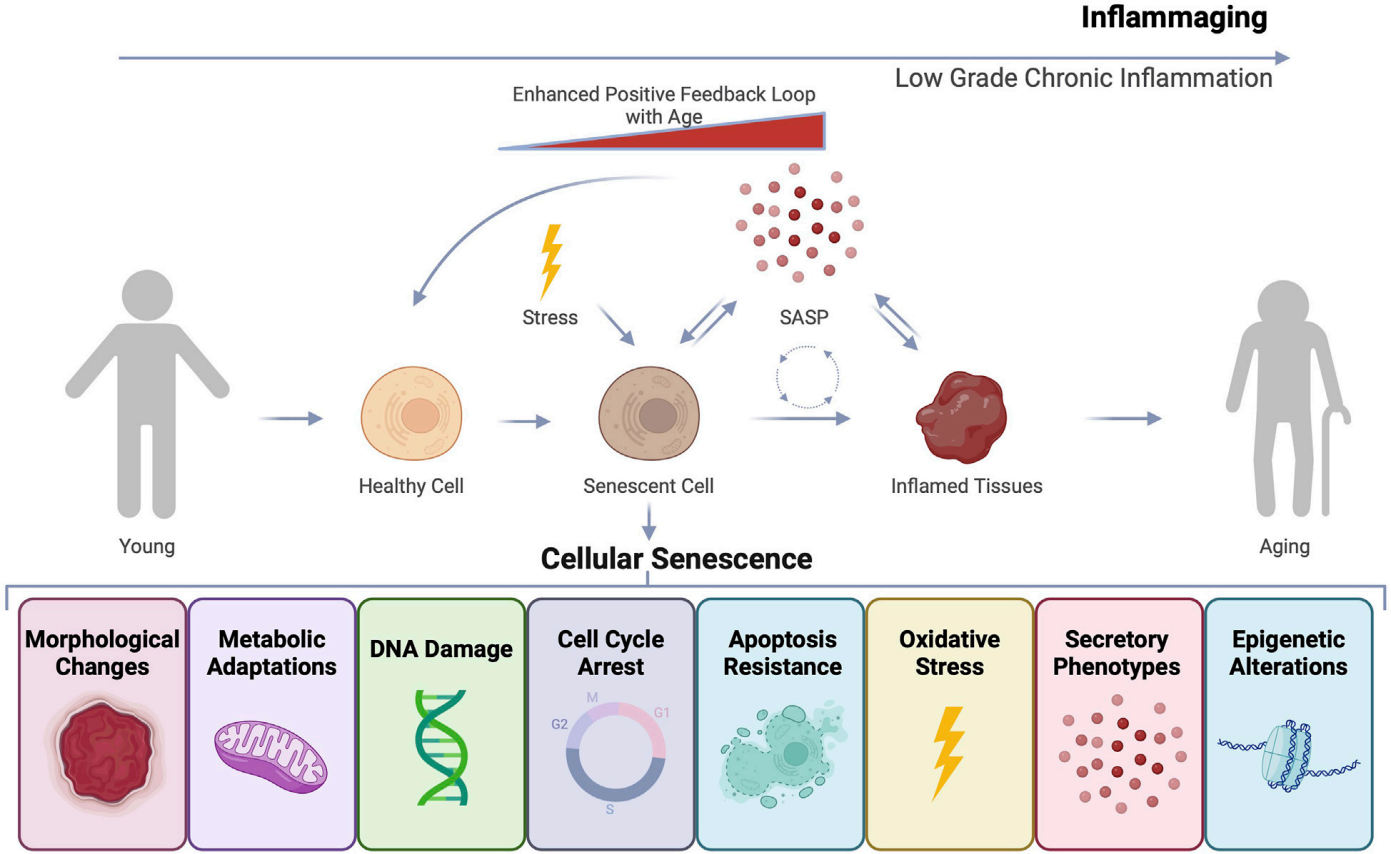
Cellular senescence and inflammaging as drivers of age-related dysfunction. Cells undergoing stress can undergo various cell fates including transformation, apoptosis, or cellular senescence. Senescent cells can exhibit diverse features such as morphological and molecular changes that result in stable cell cycle arrest and resistance to apoptosis, ensuring their continued survival. Senescent cells can also develop a secretory phenotype known as the senescence-associated secretory phenotype (SASP). The inflammatory factors that constitute SASP can induce secondary senescence and disrupt tissue, contributing to an overall state of inflammaging that accelerates the aging process.

Various cellular stressors and damage triggers can prompt distinct cell fates, among which is cellular senescence (Childs et al., 2014; Zhang et al., 2022). Cellular senescence can arise in response to several forms of intrinsic and extrinsic stimuli, including telomere shortening, DNA damage, oncogenic stress, epigenetic alterations, and mitochondrial dysfunction (Figure 1) (Gorgoulis et al., 2019). Senescent cells are known to accumulate with both age and disease (He and Sharpless, 2017; Campisi, 2013). These cells can also secrete inflammatory factors which contributes to an inflammatory state, disrupting tissue homeostasis and repair (Coppe et al., 2010; Coppe et al., 2008).

Senescent cells are quite heterogeneous and currently no universal marker of senescence exists. However, these cells can adopt several features including: stable cell cycle arrest, expression of cyclin-dependent kinase inhibitors, loss of nuclear lamina, altered metabolism and enzymatic properties, morphological changes, and secretion of soluble inflammatory factors in what is known as the senescence-associated secretory phenotype (SASP) (Table 1) (Gorgoulis et al., 2019; Zhang et al., 2022; Ogrodnik et al., 2024). SASP factors include growth factors, pro-inflammatory cytokines, chemokines, and proteases, contributing to inflammaging, a chronic, low-grade inflammation observed during aging (Figure 1) (Coppe et al., 2010). SASP can have autocrine, paracrine, and endocrine effects causing secondary senescence both proximally and distally (Zhang et al., 2022). While senescent cells play a role in normal physiological processes (de Magalhaes, 2024), their accumulation in pathogenic amounts with age and disease is detrimental. Although cellular senescence is recognized as an important tumor suppressor mechanism, in some contexts senescent cells can be pro-tumorigenic likely through adopting the SASP phenotype (Coppe et al., 2010).

**TABLE 1:** Characteristics of senescent cells.

Changes	Features
Morphological	↑ Cell and nuclei size, vacuolization
Enzymatic Activity	↑ β-galactosidase activity and lysosomal content
Cell Cycle Arrest	↑ p53, p21^CIP1^, p16^INK4a^; ↓ Ki67
Cell Surface Markers	↑ DDP4, ICAM1, NOTCH, uPAR
Nuclear	↑ DDR (ɣH2AX), TAF, SAHF, H3K9me3, H3K27me3, LINE-1 ↓ LaminB1
Cell Surface Markers	↑ DDP4, ICAM1, NOTCH, uPAR
Anti-Apoptotic Pathways	↑ BCL2L1, EFNB1/3, PI3K, SERPINB2
Cytoplasmic and Organelle	↑ CCFs and mtDNA
Senescence Associated Secretory Phenotype Factors	↑ IL-1⍺/β, IL-6, GDF15, IFNɣ, MCP-1, MMPs, TGFβ, TNF⍺, VEGF

CCF, Cytoplasmic Chromatin Fragment; DDR, DNA Damage Response; IL, Interleukin; MMP, Matrix Metalloprotease; SAHF, Senescence-Associated Heterochromatic Foci; TAF, Telomere-Associated Foci.

**FIGURE 2:**
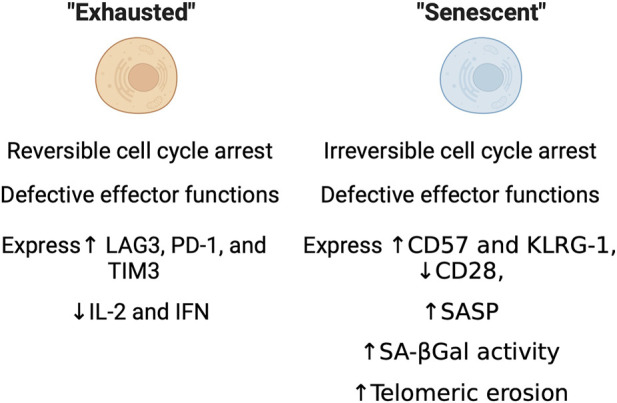
Immunological characteristics of exhausted and senescent cells. Functional, molecular, and enzymatic features as characterized through standard immunological methods.

During immunosenescence, T cells can undergo multiple dysfunctional states including: anergy, exhaustion and senescence (Crespo et al., 2013). Hypo-responsiveness to stimulation is a classic feature of anergic T cells, which are proposed to play a role in peripheral tolerance and protection against autoimmunity. Anergic T cells are characterized by low or no IL-2 production and the inability to activate and proliferate in response to exposure to antigens even in the presence of full co-stimulation (Schwartz, 2003; Crespo et al., 2013). With age, chronic infection, or disease can cause exhausted T cells to accumulate. Unlike anergic T cells, exhausted T cells lose effector function and fail to respond to stimuli (Wherry and Kurachi, 2015). Exhausted T cells are long-lived, undergo cell cycle arrest that can be reversed, and express inhibitory receptors like PD-1, CTLA, and others, and have defects in effector function (Figure 2) (Blackburn et al., 2009; Sakuishi et al., 2010; Zhou et al., 2011). T cell exhaustion is distinct from T cell senescence, and made all the more confusing as some there can be some degree in overlap of features (i.e., cell cycle arrest, expression of PD-1 in some “senescent” T cells) between both groups of cells. Immunologists originally characterized T cell senescence by phenotypic changes that included the loss of the costimulatory marker CD28, irreversible cell cycle arrest and markers associated with it (CD57 and KLRG1), impaired killing and telomeric erosion (Figure 2) (Adibzadeh et al., 1995; Effros, 1998; Appay et al., 2000). Commonly used molecular markers of senescence have been found to be present in aged T cells, but the overlap of these markers with cell surface markers or phenotypic changes commonly used by immunologists is still under investigation. Recent work has shown that common markers of dysfunction including senescence-associated β-galactosidase (SAβGal) activity at pH 6.0, elevated levels of p16^INK4a^/p21^CIP1^, SASP, and telomeric dysfunction, are present in aged human CD8^+^ T cells (Martinez-Zamudio et al., 2021). Therefore, further characterization of immune cells, their senescence status, and functional changes with age is warranted.

As we age, our immune systems can aberrantly produce persistent low-grade, chronic, and systemic inflammation in non-pathogenic or “sterile” conditions referred to as “inflammaging” (Franceschi et al., 2000; Franceschi and Campisi, 2014). Aged individuals frequently exhibit this pro-inflammatory state characterized by elevated levels of pro-inflammatory markers within cells and tissues (Franceschi et al., 2000; Franceschi et al., 2018). Inflammaging can affect both the innate and adaptive immune responses and multiple molecular mechanisms can drive this inflammation, including cellular senescence, mitochondrial dysfunction, defective autophagy, inflammasome activation, DNA damage, and changes in the microbiome (Figure 1) (Ostan et al., 2008; Goronzy and Weyand, 2013; Shen-Orr et al., 2016; Thevaranjan et al., 2017; Pawelec et al., 2020; Karakaslar et al., 2023). Inflammaging is theorized to contribute to the development of chronic age-related conditions such as cancer, cardiovascular disease, diabetes, frailty, neurodegeneration, and osteoarthritis (Franceschi and Campisi, 2014).

Understanding inflammaging is essential for exploring its broader implications, especially concerning its effects on adaptive immune responses. Immunosenescence, distinct from cellular senescence, refers to age-related detrimental changes in the immune system (Pawelec et al., 2002; Pawelec, 2012; Pawelec et al., 2020). Immunosenescence, along with inflammaging, contributes to aging and the development of age-related diseases. Both processes affect virtually every aspect of immune function: impaired vaccination efficacy, T cell differentiation and exhaustion, enhanced susceptibility to infections and increased risk of complications from infections (Fulop, 2009; Goronzy and Weyand, 2013; Weyand and Goronzy, 2016). Investigating these age-related changes is crucial for fully appreciating the consequences of inflammaging on various facets of immune function and its potential role in shaping responses to infections and vaccinations.

### Immunosenescence: a cauldron of deleterious processes

**FIGURE 3:**
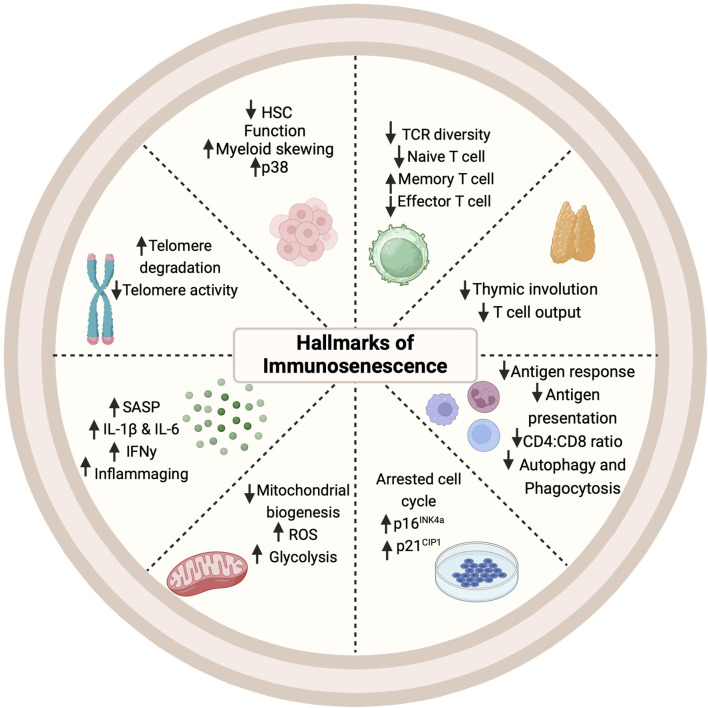
Hallmarks of immunosenescence. Cellular, molecular, and phenotypic changes occur in multiple immune cell types with the onset and progression of immunosenescence. HSC: Hematopoietic Stem Cell; IFN: Interferon; IL: Interleukin; ROS: Reactive Oxygen Species; SASP: Senescence-Associated Secretory Phenotype; TCR (T Cell Receptor).

Immunosenescence, the deterioration of the immune system with age, is characterized by thymic involution, hematopoietic stem cell (HSC) dysfunction, a disrupted naïve/memory ratio in T and B cells, inflammaging, accumulation of senescent cells, impaired responses to new antigen, mitochondrial dysfunction, genomic instability, and stress responses (Figure 3) (Liu et al., 2023). This progressive age-related decline in immune function is intricately linked to cellular senescence and chronic inflammation (Franceschi et al., 2000; Pawelec et al., 2020; Liu et al., 2023). Senescent cells can typically be cleared by immune cells and but when immunosurveillance fails, the burden of senescent cells increases, accelerating aging and shortening both health span and lifespan (Campisi and D’adda Di Fagagna, 2007; Zhang et al., 2022). Evidence suggests that the clearance of senescent cells can alleviate senescence-associated diseases and enhance T cell proliferation (Palacio et al., 2019). Beyond cellular senescence, metabolic alterations, stem cell depletion, and chronic inflammation, can also drive immunosenescence and create an immunosuppressive environment (Liu et al., 2023). The interplay between inflammation and immunosenescence also stimulates myelopoiesis (myeloid skewing) and enhances the presence of immunosuppressive cells, particularly regulatory T cells and M2 macrophages (Beerman et al., 2010; Pang et al., 2011; Ross et al., 2024). Deeper mechanistic understanding of inflammaging, cellular senescence (both in the immune compartment and the parenchyma), and immunosenescence is necessary for developing interventions to combat age-related chronic diseases.

### Age-related changes in innate immunity

Innate immunity comprises phagocytic cells, such as macrophages, dendritic cells (DCs), granulocytes (e.g., neutrophils), and innate lymphocytes like natural killer (NK) cells. These cell types mount an immediate, non-specific response to viral, bacterial, and fungal pathogens. These cells play an important role in controlling the initial stages of infection by inhibiting viral replication and spread (Carpenter and O'Neill, 2024). Immunosenescence impacts virtually every innate immune cell type (Table 2). Age-driven changes begin at the HSC level in the bone marrow (Pang et al., 2011), leading to a bias toward the myeloid lineage over lymphocyte differentiation (Muller-Sieburg et al., 2004), influenced by inflammatory cytokines like IL-6 and IL-1β (Frisch et al., 2019; Helbling et al., 2019). Beyond the differentiation bias aged HSCs are also known to have an inferior potential for immune reconstitution, engraftment, and self-renewal compared to younger HSCs (Morrison et al., 1996; Dykstra et al., 2011).

**TABLE 2:** Age-related changes in immune populations.

Stem cells	
Hematopoietic Stem Cells	↓ Overall cell number, ↓ lymphoid and ↑ myeloid differentiation, ↑ DNA damage, impaired proteostasis

Cells of innate immunity	
Dendritic Cells	↓ Plasmacytoid DCs, ↓ TLR function, ↓ IFNɣ, ↑ pro-inflammatory cytokine production
Macrophages	↓ TLR function, ↑ IL-10, ↑ pro-inflammatory cytokine, ↓ phagocytosis, impaired migration
Neutrophils	↑ counts in tissues, impaired chemotaxis, ↓ superoxide generation, ↓ phagocytic activity, ↑ susceptibility to apoptosis
Natural Killer Cells	↓ cytotoxicity, ↓ cytokine production, altered pattern of NK cell receptors, ↑ ROS production

Cells of adaptive immunity	
T Cells	↓ naïve cells, ↓ CD28, ↓ IL-2, ↑ pro-inflammatory cytokine, ↑ Th_1_ and Th_17_ CD4^+^ subsets, ↑ terminally differentiated cells, ↑ inhibitory markers, ↓ effector function, ↓ TCR diversity, loss of self-tolerance
B Cells	↓ naïve cells, ↓ peripheral B cells, ↑ memory cells, ↑ ABCs and long-lived PCs, ↓ antibody repertoire, ↓ high-affinity antibody production

While neutrophil numbers do not decrease with aging, their functionality is compromised. Aged neutrophils exhibit reduced chemotactic activity, which is crucial for reaching infection sites, thus slowing the resolution of inflammation (Fortin et al., 2008; Sauce et al., 2017). This delay is evident in older mice, where resolution of inflammation takes longer compared to younger mice (Shaw et al., 2013). Aged neutrophils upregulate of CXCR4, which chemotactically redirects them back to the bone marrow, potentially delaying their migration of neutrophils to infection sites and reducing the overall efficiency of the immune response (Uhl et al., 2016; De Filippo and Rankin, 2018). Despite stable neutrophil counts in healthy older adults, increased numbers and altered phenotypes of neutrophils are linked to age-related frailty and higher mortality (Fortin et al., 2008; Sauce et al., 2017; Lu et al., 2021). In aged mice, neutrophil counts may rise in circulation and tissues and are accompanied by impaired superoxide generation and phagocytic activity in immunosuppressive neutrophils (Van Avondt et al., 2023). These changes result from cell-intrinsic alterations and age-related shifts in the bone marrow environment, affecting neutrophil function more than their numbers (Tomay et al., 2018; Lagnado et al., 2021).

Natural Killer (NK) cells also show age-related effects, including reduced cytokine production (Hazeldine and Lord, 2013). With age NK cells, particularly long-lived NK cells, are known to accumulate in older individuals (Gounder et al., 2018; Brauning et al., 2022). The functional impairment of these cells is primarily due to decreased cytotoxicity and cytokine production (Pierson and Miller, 1996; Hazeldine et al., 2012; Hazeldine and Lord, 2013). Decreased cytotoxic NK cell subtypes are associated with an increase in viral infections and cancers (Brauning et al., 2022). Recent findings suggest that NK cells have broader functions beyond their antiviral defense. Changes in cytokine release by NK cells in older individuals further contribute to immune alterations and the accumulation of senescent cells, weakening the adaptive immune system (Tarazona et al., 2020).

Dendritic cells (DCs) are an essential component of the immune system and undergo significant changes with age, including alterations in subsets, activation, and pathogen response. Aging leads to reduced plasmacytoid DCs (pDCs) and CD141^+^ myeloid DC (mDC) subsets, which can impair T cell function (Agrawal et al., 2017). While tissue DC subsets show limited changes, increased maturation of DCs suggests heightened activation and migration with age (Granot et al., 2017). Defective Toll-like receptor (TLR) function is evident in both pDCs and mDCs with aging, compromising their ability to combat infections (Panda et al., 2010). Specifically, pDCs exhibit reduced interferon secretion, contributing to increased susceptibility to viral infections in older individuals (Swiecki and Colonna, 2015). While mDCs display decreased pro-inflammatory cytokine production, monocyte-derived dendritic cells (MoDCs) from aged subjects show increased pro-inflammatory cytokine secretion and impaired production of the anti-inflammatory cytokine IL-10 (Agrawal et al., 2013). Additionally, aged MoDCs exhibit impaired type I and III interferon production in response to viral stimuli, compromising antiviral defense and altering immune responses in older individuals (Mordstein et al., 2010).

Macrophages exhibit diverse functions crucial for maintaining tissue homeostasis, combating infections, and promoting tissue repair. They serve as pivotal regulators of the adaptive immune response through antigen presentation and activation of B and T cells (Bowdish et al., 2007; Wynn et al., 2013). Additionally, they contribute to tissue maintenance by scavenging apoptotic cells *via* efferocytosis and secreting growth factors essential for tissue development and repair (Wynn et al., 2013; Doran et al., 2020). However, aging significantly impacts the functionality of macrophages. Aging induces changes in cytokine secretion by macrophages upon stimulation of pattern recognition receptors like Toll-like receptors (TLRs) (Fitzgerald and Kagan, 2020). Studies consistently show that macrophages derived from aged mice exhibit diminished secretion of IL-1β, IL-6, IL-12, and TNFα in response to various TLR ligands compared to those from young mice. This reduction in pro-inflammatory cytokine secretion is often accompanied by increased IL-10 production, indicative of a shift toward an anti-inflammatory phenotype (Dunston and Griffiths, 2010). Additionally, aged macrophages display heightened secretion of prostaglandin E2 in response to lipopolysaccharide stimulation, further contributing to the altered cytokine profile associated with aging (Chen et al., 2022). These alterations in cytokine secretion patterns may impair the immune response to pathogens and contribute to age-associated chronic inflammation (Franceschi et al., 2018). Furthermore, aging compromises the phagocytic function of macrophages, impairing their ability to clear debris and pathogens, which is critical for tissue repair and regeneration processes (Yarbro et al., 2020). These age-related changes in macrophage functionality contribute to impaired wound healing, delayed tissue repair, and increased susceptibility to infections in aged individuals.

### Age-related changes in adaptive immunity

The adaptive immune system, composed of B and T cells, plays an essential role in clearing pathogens and mounting robust responses to vaccination. These cell types orchestrate a targeted defense against threats to the body. B cells are vital components of cellular and humoral immunity by producing antibodies, presenting antigens, and regulating T cell functions (Lee et al., 2022). Aging can introduce significant changes in B cell development (Table 2), affecting both intermediate and mature stages (Frasca et al., 2020). Older individuals typically have decreased peripheral B cells, but an increase in pro-inflammatory B cells driven by elevated signals such as CD40L, IFNɣ, and IL-21 (de Mol et al., 2021; Nickerson et al., 2023). Aging can also cause reduced expression of molecules in B cells crucial for immunoglobulin class-switch recombination and somatic hypermutation (Frasca et al., 2020). Impairment of these two key processes for generating immunological diversity negatively impacts the production of high-affinity antibodies, impairing the immune system’s ability to generate productive antibodies responses in aged individuals (Frasca et al., 2020). Consequently, the aged immune system may produce antibodies with reduced effectiveness, potentially increasing susceptibility to bacterial and viral infections, including diseases like COVID-19 (Kline and Bowdish, 2016). Diminished antibody production with age could be influenced by a number of mechanisms including: lymphopenia, impair cytokine production in DCs and other antigen-presenting cells (Banchereau et al., 2000; Panda et al., 2010; Metcalf et al., 2017), downregulation of co-stimulatory marker CD28 in aged T cells (Vallejo, 2005), age-related defects in class-switch recombination that diminish the production of secondary isotypes (Frasca et al., 2011), and lastly age-related shifts in B cell population and intrinsic defects like increased production of TNF⍺ by aged B cells that suppresses their function (Frasca et al., 2014; Frasca et al., 2017; Frasca et al., 2020). Strategies to improve vaccination efficacy in the elderly include high-dose vaccinations, use of multivalent vaccines to provide broader coverage of antigenic-variable pathogens, use of specific adjuvants to elicit better responses in DCs, and reductions in chronic inflammation and immunosenescence (discussed later in the manuscript) (Hou et al., 2024).

Studies have shown that aging significantly impairs precursor B cell production in both the bone marrow of aged mice and humans (Miller and Allman, 2003; Frasca et al., 2020). This reduction is attributed to changes in the bone marrow microenvironment, including decreased levels of the pro-B cell-survival cytokine IL-7 and a shift in HSC differentiation towards myeloid cells instead of lymphocytes, a process known as myeloid skewing (Labrie et al., 2004). While the absolute number of peripheral B cells declines with age in humans, aged mice maintain mature splenic B cell numbers due to the emergence of autoreactive and age-associated B cells (ABCs) (Ratliff et al., 2013; Cancro, 2020; de Mol et al., 2021). ABCs, a unique subset observed in both mice and humans, are characterized by specific markers and signal transduction pathways (Cancro, 2020). These cells, known for producing characterized autoantibodies, are closely linked to autoimmune diseases like lupus and rheumatoid arthritis, and more prevalent in aged individuals (Wehr et al., 2004; Adlowitz et al., 2015; Claes et al., 2016). Understanding the complex relationship between aging and B cell dynamics could further shed light on the complexities of age-related changes in the adaptive immune system.

T cells, and the effects of aging on them (Table 2), have been extensively studied due to their crucial roles in the immune response as both effectors and regulators (Mittelbrunn and Kroemer, 2021). Originating from HSCs, T cells mature in the thymus and differentiate into various subsets such as helper T cells (Th), cytotoxic T lymphocytes (CTLs), and regulatory T (Treg) cells (Sun et al., 2023). Thymic involution, a natural deterioration of the thymus structure with age and a key feature of immunosenescence. This process causes a diminished output of naive T cells and leads to functional deficiencies such as a reduced peripheral T cell repertoire and IL-2 production, resulting in a compensatory clonal expansion of memory T cells (Goronzy and Weyand, 2019; Elyahu and Monsonego, 2021).

Senescent T cells, characteristic of aging, show a decline in essential signaling molecules like CD21 and CD28, reduced IL-2 production, and increased secretion of pro-inflammatory cytokines (Callender et al., 2018; Goronzy and Weyand, 2019). These senescent T cells contribute to inflammation by interacting with other immune cells or directly affecting target tissues, leading to tissue damage and aging-related pathologies (Carrasco et al., 2022). Studies in mice have found that senescent immune cells, including T cells, were shown to not only prematurely age the immune system but also cause premature aging in non-lymphoid organs (Desdin-Mico et al., 2020; Yousefzadeh et al., 2021; Gabandé-Rodríguez et al., 2024). Th cells in aged individuals also exhibit a shift towards effector memory cells, accompanied by increased cytokine secretion and alterations in key receptors (Zhang et al., 2021). Th1 and Th17 cell subsets become dominant with age, correlating with increased inflammation and compromised immunity (Zhang et al., 2021; Merino et al., 2021). The accumulation of senescent cells was recently reported to cause CD4^+^ T cells to differentiate into cytotoxic T lymphocytes (CTLs) (Elyahu et al., 2024). The primary transcription factor involved in their differentiation is Eomes (Soto-Heredero et al., 2023), which plays a fundamental role in the generation of these lytic molecules and cytokines (Ovadya et al., 2018; Thelen et al., 2023). CTLs are indispensable for defense, but demonstrate impaired proliferation, reduced cytotoxicity, and a decrease in naive cell markers (CD45RA and CD27) with age (Soto-Heredero et al., 2023; Zhang et al., 2023; Hasegawa et al., 2023; Gabandé-Rodríguez et al., 2024). Aged CTLs also exhibit cellular senescence characteristics as well such as dysfunctional telomeres and high levels of SAβGal activity and p16^INK4a^ expression, elevating disease risk (Martinez-Zamudio et al., 2021; Yousefzadeh et al., 2021; Elyahu et al., 2024). Eliminating CD4 CTLs in late aging stages led to increased accumulation of senescent cells, profound physical deterioration, and decreased lifespan. Remarkably, senolytic treatment to reduce the burden of senescent cells effectively halted this differentiation process (Elyahu et al., 2024).

Tregs are responsible are for modulating the immune response, but with age their population increases despite thymic involution, while their function either remains stable or increases, due to the higher expression of Foxp3 (Jagger et al., 2014; Rocamora-Reverte et al., 2020). Murine studies also showed impaired immunosuppressive responses in aged Tregs (Palatella et al., 2022). The transfer of CD25^+^ Tregs from aged mice into young recipients resulted in lower suppression of hypersensitivity responses compared to the transfer of Tregs from young mice (Dieckmann et al., 2001). Conversely, Tregs from aged mice showed reduced inhibition of responder cell proliferation and decreased production of the anti-inflammatory cytokine IL-10 (Raynor et al., 2012). Also, the increased presence of Treg cells in older individuals can be explained by the age-related accumulation of mature Tregs at the expense of naive CD4^+^ T cells (Thiault et al., 2015). Furthermore, activated Tregs that acquire an effector/memory phenotype can migrate back to the thymus, hindering the generation of new thymic Tregs (Raynor et al., 2012). This competition for IL-2 results in an aging Treg pool favoring an effector/memory phenotype, diminishing clonal diversity (Weist et al., 2015). This selective suppression may allow specific pro-inflammatory cells to remain active in aged hosts. Lastly, age-related changes in genes related to leukocyte activation reduce the ability to recognize new pathogens, impacting responses to vaccination and increasing infection risks in the old (Yang et al., 2020a). Despite significant progress, challenges remain in fully understanding the intricate functions of T cells and how they are lost in immunosenescence.

### Impact of gut microbiota health on immune aging

Gut dysbiosis, an imbalance in the gut microbiota composition, can significantly impair the immune system and accelerate immune aging. The gut microbiota plays a crucial role in shaping immune resilience by influencing the development and function of the immune system. Through interactions with host immune cells and the intestinal epithelium, gut microbes help maintain immune homeostasis and defend against pathogens (Dantzer et al., 2018; Zheng et al., 2020). Commensal bacteria in the gut produce various metabolites and microbial-associated molecular patterns (MAMPs) as part of their normal metabolic activities (Chenard et al., 2020). These molecules interact with the host immune system through TLRs on immune cells. Activation of these receptors by MAMPs modulates immune cell responses, including maturation, activation, and function (Chenard et al., 2020; Zheng et al., 2020). Dysbiosis disrupts this critical interaction, leading to immune dysregulation, increased susceptibility to infections, and chronic inflammatory diseases (Santana et al., 2022). Conversely, a diverse and balanced gut microbiome supports robust immune responses and reduced inflammation, highlighting the importance of gut health in maintaining immune resilience (Zheng et al., 2020). Gut health can be influenced by perinatal effects, maternal components, socio- and psychological factors that can occur early on but have life-long effects (Salazar et al., 2023). Interventions such as probiotics, prebiotics, fecal microbiome transplantation, and dietary changes are promising strategies to restore gut balance and mitigate age-related immune dysfunction, offering potential therapeutic avenues for optimizing immune health (Salazar et al., 2023). Probiotic treatment in the SAMP8 mouse model of accelerated aging reduced levels of myeloid inflammation and improved memory deficits (Yang et al., 2020b). Fecal microbiome transplantation containing *Akkermansia muciniphilia*, known to improve immune function and metabolic homeostasis (Plovier et al., 2017; Schneeberger et al., 2015), into two different mouse model of Hutchinson-Guilford Progeroid Syndrome was able to correct gut dysbiosis, improve age-related phenotypes and extend lifespan (Barcena et al., 2019). While lack of exercise and poor diet is known to cause microbiota shifts, gut leakiness and subsequent inflammation (Brooks et al., 2023), caloric restriction has shown beneficial changes in gut microbiome including increased levels of naïve T cells, and a reduction in exhausted immune cell populations (Sbierski-Kind et al., 2022). Regular physical activity is known to be maintain a beneficial flora, reduce inflammation and improve immune function in aged individuals (Duggal et al., 2018; Koblinsky et al., 2023).

### Impact of sex-based differences on immune aging

Recent research has shed light on the significant role of sex in immune aging, revealing distinct differences between males and females in the context of aging-associated immune alterations (Mkhikian et al., 2022; McGill and Benayoun, 2022). A comprehensive study investigated age-related changes in naive T (Tn) cells and found notable sex-dimorphic variations in Tn function, with older females exhibiting diminished Tn function compared to males across both mice and humans (Mkhikian et al., 2022). This study underscores the need to consider sex-specific factors in understanding immune aging and highlights the importance of tailored therapeutic approaches. Moreover, the study elucidates the potential influence of extrinsic factors, such as IL-7 signaling, on the observed sex differences in immune aging, offering valuable insights into the intricate interplay between biological sex and aging-related immune dysfunction (Mkhikian et al., 2022). One study found a correlation between higher innate immune cytokines and disease progression in females and poorer T cell responses with disease progression in males (Takahashi et al., 2020). Variations were observed in cytokine/chemokine levels, with higher IL-8 and IL-18 in male patients at baseline and elevated CCL5 levels longitudinally. Moreover, differences in monocyte populations and T cell activation were noted, with male patients showing lower T cell counts and higher levels of CD14^+^CD16^−^ non-classical monocytes compared to female patients (Takahashi et al., 2020). These studies highlight the necessity for sex-specific considerations in therapeutic strategies and further investigations to elucidate sex-dependent mechanisms underlying immune function and disease susceptibility.

Furthermore, older individuals have long been perceived to respond poorly to vaccination, casting doubts on the overall efficacy of immunization in old age. Age-related alterations in innate and adaptive immunity render older adults more vulnerable to infections like influenza, COVID-19, and bacterial pneumonia (Pinti et al., 2016). COVID-19 mortality rates soar among the aged, with 80% of the deaths occurring in individuals over the age of 65 (Powell et al., 2020). Sexual dimorphic effects around vaccination efficacy are present in older individuals (Yanicke and Ucar, 2023). A recent study found that older men mount weaker responses to some pneumococcal vaccines (Ravichandran et al., 2024). Despite vaccination recommendations, age-related immune changes diminish vaccine efficacy, underscoring the need for a deeper understanding of immune aging to enhance older adults’ health span and resilience against infectious diseases (Ciabattini et al., 2018).

### Impact of social determinants on immune aging

Inherently, extrinsic stress has also been shown to affect immune aging. Chronic psychological stressors have been linked to an increased risk of developing common colds upon exposure to cold viruses (Cohen et al., 1998; Dantzer et al., 2018; Dantzer, 2018). This association arises from chronic stress interfering with the body’s ability to regulate the immune system’s production of inflammatory chemicals. While acute stress typically increases cortisol levels, which can reduce inflammation, chronic stress may lead to glucocorticoid resistance, wherein immune cells become insensitive to cortisol, thereby perpetuating inflammation (Miller et al., 2002). This failure in immune regulation may contribute to heightened susceptibility to infections like the common cold, emphasizing the intricate interplay between stress and immune resilience. Additionally, patients with major depressive disorder and post-traumatic stress disorder often exhibit elevated levels of inflammatory markers, including IL-6, suggesting a link between systemic inflammation and stress-related disorders (Baune et al., 2012; Newton et al., 2014). A study investigating the relationship between social stress and immunity found that individuals experiencing chronic stress, a stressful event, or everyday discrimination had reduced naïve T cells while the population of exhausted T cells increased (Klopack et al., 2022). Although the injection of proinflammatory cytokines can induce symptoms resembling depression, establishing a causal relationship between peripherally derived cytokines and stress-related disorders requires further investigation (Dantzer et al., 2008). In animal models, chronic social subordination leads to depression-like behavior in susceptible models, while resilient models exhibit resistance to such behavioral changes (Russo and Nestler, 2013). These findings suggest that preexisting differences in the sensitivity of the peripheral immune system may influence an individual’s vulnerability or resilience to social stress (Russo and Nestler, 2013). Other studies have found increased markers of immunosenescence (reduced thymic function, lower naïve CD8^+^ T cells, diminished CD4^+^:CD8^+^ T cell ratios, and increased levels of terminally differentiated effector memory CD4^+^ T cells that re-express CD45RA (T_EMRA_), *etc.*) strongly correlated with multiple social determinants including racial/ethnic disparities, educational attainment, socioeconomic status, housing, and income (Noppert et al., 2023; Aiello et al., 2016; McClure et al., 2018).

### Immune resilience

Immune resilience (IR) is a critical component of fully understanding immunosenescence. Immune resilience refers to the ability of the immune system to maintain or quickly restore its functions, thus promoting disease resistance and controlling inflammation during infectious diseases and other inflammatory stressors (Lee et al., 2021). A recent study analyzed the concept of immune resilience and its profound implications for longevity and overall health outcomes were analyzed. The metrics utilized in the study to measure immune resilience included Immune Health Grades (IHGs) and transcriptomic profiles (Ahuja et al., 2023). IHGs were introduced as a novel classification system aimed at quantifying immune resilience by evaluating the equilibrium between infection-fighting CD8^+^ and CD4^+^ T cells. These grades, ranging from I to IV with higher IHG grades indicated a more favorable balance of CD8^+^ and CD4^+^ T cells, with lower grades reflecting optimal immune resilience. This classification system not only captures variations in T-cell populations but also offers clinical relevance, distinguishing between different immune states during conditions such as acute COVID-19 or advanced HIV disease. Notably, patients with high levels of CD4^+^ T cells and low levels of CD8^+^ T cells (IHG-I profile) during SARS-CoV-2 and HIV infections were the least likely to develop severe COVID-19 and AIDS (Ahuja et al., 2023). Importantly, IHGs served as valuable indicators of immune health and were associated with superior health outcomes and longevity.

Complementing IHGs, transcriptomic profiles provided insights into the molecular mechanisms underlying immune resilience and its association with survival and mortality risks. These profiles delineated distinct gene expression patterns, such as survival-associated signature (SAS)-1 and mortality-associated signature (MAS)-1, offering deeper insights into the correlation between immunocompetence and inflammation. Transcriptomic analysis involved high-throughput sequencing and bioinformatics tools to identify genes and pathways associated with immune resilience. SAS-1, characterized by genes associated with immune cell function, showed higher expression in individuals with lower all-cause mortality risks during acute COVID-19 and in the general population (Ahuja et al., 2023). Together, IHGs and transcriptomic profiles provided a comprehensive approach to measuring immune resilience, encompassing both cellular and molecular aspects of the immune response. These metrics offered valuable insights into the dynamics of immune health and its implications for longevity and overall health outcomes (Brodin and Davis, 2017). Further research and validation of these metrics hold the potential to inform personalized interventions aimed at enhancing immune resilience and mitigating age-related immune dysfunction. The study represents a significant advancement in understanding immunosenescence and highlights the importance of maintaining robust immune health as individuals age. By identifying a subset of aged individuals who maintained higher levels of resilience, the study challenges conventional notions of immune decline as an inevitable consequence of aging and underscores the potential for interventions to bolster immune resilience and mitigate immunosenescence.

Other methods beyond flow cytometric immunophenotyping or transcriptomic analysis for assessing immune function could be integrated to assess immune resilience in both mice and humans. T cell-specific endpoints include *ex vivo* stimulation and cytokine production, delayed type hypersensitivity using a foreign antigen like keyhole limpet hemocyanin, or cytotoxicity assays (Waldorf et al., 1968, Fulo, 2009; Mittelbrunn and Kroemer, 2021). Similar to T cells, NK cell cytotoxicity against a target cell line lacking MHCI (e.g., K562 cells) can be assessed (Brauning et al., 2022). Arguably, one of the most relevant and easiest endpoints for the assessing immune resilience is the response to vaccination and the quantification of antigen-specific antibody titer levels (Goronzy and Weyand, 2013; Ciabattini et al., 2018). While there is no standard battery of endpoints for the assessment of immune resilience, vaccine efficacy (titers), complete blood chemistries with differential counts (assessment of lymphopenia), and flow analysis (quantification of naïve and terminally exhausted cells) are the most commonly used measurements. However, future incorporation of transcriptomic analysis, especially single cell (Mogilenko et al., 2021; Terekhova et al., 2024), and the use of composite scoring systems like IHGs (Ahuja et al., 2023) could be beneficial.

Individuals with immune resilience are likely to have high immunocompetence and functionality, along with minimal background inflammation, which can potentially help buffer against the immune and systemic effects of harmful stimuli. However, reliably capturing this remains elusive. Much of the knowledge in this area is derived from disparate animal studies that lack standardized models, cross-sectional or short-term longitudinal analyses, and have limited endpoints. These factors underscore the need for longitudinal analyses to track immunological changes throughout the life course of individuals and in response to challenges that can accelerate biological aging (such as infections, injuries, stress, and social determinants). Evaluating immunological aging in both short-lived individuals (e.g., those with progeroid syndromes or inborn errors of immunity) and long-lived individuals (such as the “wellderly” and centenarians) could provide valuable insights into the underlying biology of immune aging and resilience. While previous studies suggest that immune variation is largely driven by non-heritable factors (Brodin et al., 2015; Brodin and Davis, 2017), investigating heritable influences on immunity is still worthwhile and presents an opportunity to identify novel therapeutic targets.

Current studies on immune resilience have been largely limited to basic immunophenotyping of common immune populations, cytokine analyses, or transcriptomic studies on leukocytes. Although cytokine analysis can provide information on the inflammatory background and immunophenotyping can describe population shifts, additional functional studies are needed to properly assess immunocompetence and how it changes in response to age, immune challenges, or other forms of stress. Furthermore, these endpoints need to be accessible, practical, and cost-effective for widespread clinical deployment. These steps are critical for identifying and benchmarking biomarkers or surrogate endpoints for immune resilience that can: 1) be translated back to pre-clinical animal models to generate standardized models with translational potential, and 2) be used to more reliably assess interventions that promote immune resilience and allow for comparisons between them.

### Strategies to overcome age-related changes to the immune system

**FIGURE 4:**
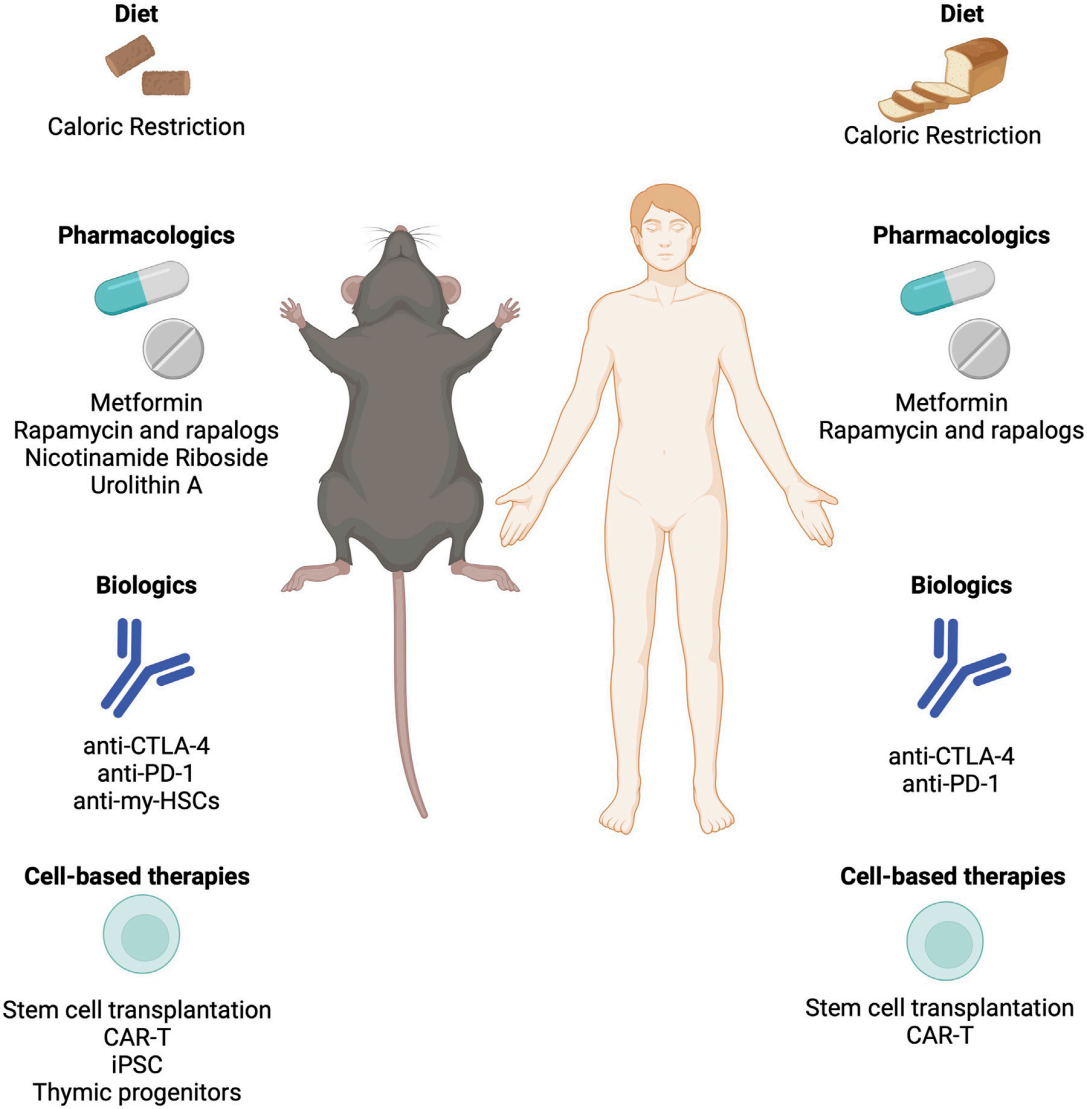
Strategies to rejuvenate an aged immune system and restore immune resilience. Interventions including biologics, cell-based therapies, and pharmacological approaches that have been demonstrated to restore immune function in mice and humans. CAR-T: Chimeric Antigen Receptor T Cell; CTLA-4: Cytotoxic T Lymphocyte Associated Protein-4; iPSC: Induced Pluripotent Stem Cells; PD-1: Programmed Cell Death Protein 1.

Research into strategies to counteract age-related declines in immunity and to test the maintenance or restoration of immune resilience is ongoing and holds promise for healthy aging (Figure 4). Geroscience-directed approaches, which target the underlying mechanisms of aging, complement novel strategies aimed at fortifying the immune system. One of the most effective gerotherapeutic interventions is caloric restriction (CR), which has been demonstrated to be beneficial in various animal models and in humans (Green et al., 2022). In addition to extending lifespan in mice and reducing cardiometabolic risk factors in humans, CR has been shown to improve immune function in aged mice and healthy humans (Asami et al., 2022; Spadaro et al., 2022). Vaccines tailored for older adults, with higher antigen concentrations or adjuvants, exhibit enhanced efficacy against viral infections like influenza (McElhaney et al., 2020). In the pursuit of rejuvenating T cell pools, three primary strategies have emerged: replacement, reprogramming, and the use of engineered T cells.

Replacement strategies involve removing dysfunctional immune cells from circulation to promote the homeostatic expansion of memory and effector T cells. Immune checkpoint inhibitors such as CTLA-4 and PD-1 are common targets for antibody-mediated depletion. A recent target has emerged is myeloid-biased HSCs, which accumulate with age in both humans and mice. Depletion of myeloid-biased HSCs with a cocktail of anti-CD62p, -CD150, and -NEO1 antibodies rebalanced progenitor pools, increased lymphocyte numbers and reduced inflammation in naturally aged mice (Ross et al., 2024). Autologous stem cell transplantation has shown success in reconstituting functional T cell pools in autoimmune diseases and hematological malignancies (Delemarre et al., 2016). Cell reprogramming using induced pluripotent stem cell (iPSC) technologies offers a promising avenue to differentiate T cells away from exhausted and senescent states, either through redifferentiation from T-induced pluripotent stem cells (T-IPSCs) or by enhancing telomerase activity to prevent telomere-dependent T cell senescence (Karagiannis et al., 2016). Restoration strategies aim to reverse thymic involution, with bioengineered thymus organoids and growth-promoting factors showing potential to rejuvenate the peripheral T cell pool and trigger *de novo* thymopoiesis (Tuckett et al., 2017). Despite challenges such as donor-specific immune tolerance and reproducing the complex thymic extracellular matrix, preclinical studies have demonstrated the effectiveness of thymic organoids in rejuvenating T cell function (Tormo et al., 2017).

The utilization of chimeric antigen receptor (CAR)-T cell therapy presents a promising strategy in combating senescence-associated pathologies. The development of “armored” CAR-T cells engineered to express additional proteins or fusion receptors offers avenues for enhancing effector function and prolonging persistence in the local microenvironment (Hong et al., 2020). Beyond oncology, CAR-T cells have been explored as potential treatments for autoimmune diseases, HIV, and cardiac fibrosis, highlighting their versatility in addressing diverse pathologies. The integration of CAR-T cells as senolytics opens new possibilities for targeted and effective therapeutic interventions against a broad spectrum of senescence-associated diseases, providing a platform for continued innovation in T cell-based therapies (Amor et al., 2020; Amor et al., 2024; Eskiocak et al., 2024; Deng et al., 2024). Use of NKG2D ligands or urokinase-type plasminogen activator receptor (uPAR) are frequent targets for senolytic CAR-T cell approaches. However, these CAR-T approaches are not without their caveats and concerns. Senescent cells can shed NKG2D ligands through the release of matrix metalloproteases (MMPs), which make up SASP. MMP-dependent cleavage of NKG2D ligands is capable of driving immunoevasion of senescent cells to promote aging (Deng et al., 2024). While the use of uPAR-specific CAR-T cells is promising (Huang and Liu, 2020), further refinement to alleviate concerns over clinical toxicity are needed (Yu et al., 2019).

Metabolic interventions, such as metformin or mTOR inhibitors, show potential for bolstering immunity and increasing resistance to infectious diseases in aging populations (Mannick et al., 2014; Barzilai et al., 2016; Mannick et al., 2018; Martin et al., 2023). The emergence of senolytics, a class of drugs designed to eliminate senescent cells, presents a promising avenue for alleviating age-related ailments, potentially including immune dysfunction (Chang et al., 2016; Camell et al., 2021; Zhang et al., 2022; Lorenzo et al., 2023). As research in this area progresses, further innovative approaches may emerge, offering new avenues to enhance immune function and extend the longevity of older adults. Metformin targets biological drivers of aging and directly influences immune function in various contexts. Metformin’s well-documented anti-inflammatory properties, demonstrated by reductions in cytokine levels across different contexts, offer significant promise in mitigating excess inflammation, irrespective of diabetes status (Hartwig et al., 2021). Additionally, metformin exhibits context-specific immunomodulatory effects on monocytes, macrophages, and other cells of the mononuclear phagocyte system, which are critical for frontline defense against infections. Studies indicate that metformin promotes an anti-inflammatory response by stimulating the transition of pro-inflammatory M1 macrophages to the anti-inflammatory M2 phenotype, thereby reducing inflammation and improving immune regulation (Jing et al., 2018). Moreover, metformin impacts T cells and adaptive immune responses by inhibiting T-cell trafficking and activation, inducing Treg polarization, and enhancing memory CD8^+^ T cell formation, among other mechanisms (Nikolich-Zugich, 2018). It also attenuates reactive oxygen species and reactive nitrogen species levels, thereby improving age-related changes in B cells and enhancing antibody responses (Nikolich-Zugich, 2018). These multifaceted effects of metformin on immune cell function underscore its potential to bolster immunological resilience in older adults, offering promising avenues for improving health outcomes in the face of viral illnesses like influenza and SARS-CoV-2.

Treatment of rapamycin, an mTOR inhibition has been shown to extend lifespan in mice (Harrison et al., 2009) and is one of the gold standards for gerotherapeutic efficacy (Mannick and Lamming, 2023). Rapamycin treatment has been shown to rejuvenate aged HSCs and allowing for effective vaccination of aged mice and enhancing their survival in response to a lethal challenge with the influenza virus (Chen et al., 2009). Using a mouse model of premature immune aging, rapamycin was able to partially rescue some of the age-related deficits in immune function and reduce markers of senescence in the immune cells of treated mice (Yousefzadeh et al., 2021). Human studies using prophylactic treatment with a rapalog in older individuals before vaccination against influenza, showed that RAD001 treatment in the study participants yielded a 20% increase in antibody titer levels as well as reduced levels of the immune dysfunction marker PD-1 (Mannick et al., 2018; Mannick et al., 2014). Specifically, inhibition of PD-1 signaling has been shown to restore T cell proliferation and effector functions such as IFNɣ production in response to antigen stimulation in aged mice (Lages et al., 2010). A recent study found that mTOR inhibition enhanced the effect of PD-1 blockade in naturally aged mice, improving the function of exhausted CD8^+^ T cells in a model of chronic infection (Ando et al., 2023). While rapamycin has long been used in the transplantation field (at much higher doses) to as an immunosuppressive agent to prevent graft-versus-host disease, it shows promise for immune rejuvenation purposes if the optimal dose and delivery schedule can be determined (Mannick and Lamming, 2023). Other gerotherapeutics such as the mitophagy activator Urolithin A or NAD precursor nicotinamide riboside have been able to restore function to aged HSCs or dysfunctional immune cells in mice (Girotra et al., 2023; Desdin-Mico et al., 2020). A better understanding of the molecular underpinnings of the pathways targeted by these interventions treat in the formation and progression of age-related immune dysfunction is needed.

Lastly, simple to enact lifestyle interventions like exercise (discussed above) or sleep hygiene can have beneficial effects on immune function (Garbarino et al., 2021). Sleep fractioning and debt has been reported to cause epigenetic shifts in HSCs and progenitor cells, eventually causing increased production of myeloid cells (McAlpine et al., 2022). Animal studies showed that improved sleep was capable of reducing aberrant myelopoiesis and reducing immune cell infiltration into the vasculature of mice (McAlpine et al., 2019). Thus while interventions to correct age-related deficits in immunity are still in development, lifestyle interventions like diet, exercise, improved sleep, and stress reduction are readily available methods to potentially improve or maintain immune function.

## Conclusion

Aging profoundly impacts the immune system, leading to various deleterious changes collectively known as immunosenescence. This phenomenon compromises innate and adaptive immunity, characterized by the reduced functionality of immune cells such as T cells, B cells, and natural killer cells (Table 2), alongside chronic low-grade inflammation termed inflammaging (Figure 1). These changes increase susceptibility to infections, diminish vaccine efficacy, and elevate the risk of age-related diseases. Immunosenescence and the subsequent loss of immune resilience is a multifactorial process that involves age-related changes in both innate and adaptive immunity. These changes are also influenced by sex, stress and other social determinants of health, and individual microbiota. The implementation of robust and standardized measurements of immune resilience is needed for both preclinical and clinical studies to assess not only the loss of resilience but also whether it can be restored through interventions discussed above. Future research should continue to investigate the intricate relationships between aging, immune function, and external influences, aiming to develop targeted therapies to mitigate the effects of immunosenescence and restore immune resilience. By fostering a deeper understanding of these mechanisms, we can improve the quality of life and extend the healthy lifespan of the aging population.
